# Medical Opinion and Movement

**Published:** 1911-09-09

**Authors:** 


### Medical Opinion and Movement.

### Vascular Transplantation.

The experimental researches of Carrel have shown the possibility of restoring the continuity of an artery partially resected, by interposing between the divided ends of the vessel a venous segment, generally taken from a corresponding adjacent vein. The operation has actually been carried out on the human subject four times. The latest case is reported by Dr. Mantelli. The patient was a man aged forty-one, and admitted to hospital at Turin for sarcoma of the middle third of the right thigh. The growth was removed. Five months later the patient complained of pain at the old site, and a tumour appeared soon afterwards, which involved the main vessels of the limb. A further operation was decided upon. In the course of the operation it was found necessary to resect 5 centimetres of the femoral artery and vein. As a sufficient collateral venous circulation was already established, it was decided to ligature the vein altogether and to restore the continuity of the artery by interposing between the divided ends of the vessel a corresponding length of the femoral vein. The arteriovenous sutures were made according to the method of Carrel. The results of this operation were excellent. The arterial circulation was in no way interrupted, and at the end of a fortnight the arterial pulsations of the femoral artery could be felt as well in that limb as in the corresponding part of the other limb.

### Trachoma and Solid Carbonic Acid.

The treatment of all manner of superficial lesions, nævi, angioma, etc., by the application of solid carbonic acid is already well-known. Some little time back Dr. Harman reported a case of granular conjunctivitis cured by this method. More recently, Dr. Montagu Harston, of the Tung Wa Hospital at Hong Kong, inspired by this report, has successfully treated fifty cases of trachoma. The method of application is as follows: Having shaped up some carbonic acid snow into the form of a pencil, the operator places himself behind the patient and everts the eyelids in the usual manner. Holding the everted eyelids with the fingers of the left hand the carbonic acid pencil is applied to the conjunctiva with the right, taking care to avoid any contact with the cornea. The patient is instructed to look downwards so that the pencil may be applied to the oculo-palpebral cul-de-sac. Having made the application on the conjunctiva of the upper and lower lids, they should be kept everted for a few moments, in order to avoid any excess of carbonic acid deposited on the lids coming in contact with the cornea. The applications cause less pain than copper sulphate, silver nitrate, or other caustics of this nature, and it passes off in a couple of minutes. The duration of the application should not exceed fifteen seconds at first, but later it may be prolonged to twenty or even thirty seconds, and it should be repeated once a week. The advantages claimed for the treatment are that the course of the affection is considerably shortened and the cicatrices are much less marked than with the usual caustics. The treatment is especially applicable to cases that have become chronic, with little secretion and a tendency to develop cicatricial tissue.

### Treatment of Tetanus.

In the *Berliner Klinische Wochenschrift* appears a report on a large number of cases of tetanus treated by Dr. Bacelli's method of subcutaneous injection of carbolic acid. Of ninety-four cases of severe tetanus collected from medical literature (mostly Italian) only two proved fatal. Of thirty-eight very severe cases, sixteen died. From these latter Dr. Bacelli excludes eleven cases, because the doses administered were wholly insufficient. This reduces the mortality of the latter series to 18.5 per cent. Considering that the mortality of severe cases of tetanus is 100 per cent., in ordinary circumstances, these figures would appear to be highly satisfactory. Dr. Bacelli uses a 2 to 3 per cent. watery solution of carbolic acid, which is injected subcutaneously. He commences with very small doses, but as soon as the proper tolerance of the patient has been established by examination of the urine, the dose should be rapidly increased until the patient takes 1 to 1½ gramme in the course of twenty-four hours. According to the author, tetanus patients tolerate carbolic acid in surprisingly large doses, and he is led to formulate the axiom that the toleration is directly proportionate to the severity of the case.

### Otitis Interna Following Mumps.

Internal ear deafness as a complication of mumps is now fortunately extremely rare; but a generation ago it seems to have been much more common. Thus in 1885 Pearce reported forty cases from his own practice. Nowadays the combination is distinctly uncommon, and as a rule it is only aural specialists who have opportunities of meeting the condition. Most of the authors of text-books recommend very strongly the administration of pilocarpine, and Yearsley even states that no case of labyrinthine deafness due to mumps should be allowed to go uncured. In the *Medical Chronicle* Mr. J. A. Jones relates the history of a child, aged eight, who, while recovering from an attack of mumps, suddenly became quite deaf in both ears. He first saw the patient nine days after the onset of the mumps, and three days after the onset of deafness. There was some vertigo, but no tinnitus and no nystagmus. The middle ear was unaffected; pilocarpine injections subcutaneously were begun at once and continued for three weeks. On the fifth day the vertigo disappeared, but the deafness remained. Pilocarpine and quinine were then given by the mouth for a fortnight, and after that strychnine was tried, but without avail. As a rule the condition is unilateral, but in this case it was bilateral, and permanent complete deafness has resulted.

### The Action of Sulphur Compounds.

A very interesting train of thought is suggested by some researches into the action of sulphur compounds which Dr. C. O. Jones describes in the *Bio-Chemical Journal*. The investigation dealt first of all with the result of administering soluble sulphates hypodermically; it was thus proved, as had been supposed before but denied by some observers, that when thus given no purgative effect follows. A complete research was then undertaken, and the author finds that sulphates, hyposulphites, sulphites, and sulphides interfere with oxidation processes in the cells; the sulphates do this principally by preventing exchange between the blood-stream and the cells. This action disappears when the amount of sulphate is reduced, and there then follows a stage of stimulation, the sulphate causing a very marked diuresis, sometimes accompanied by the execretion of more sulphates than were injected into the body together with the amount found in the food. Purgation is not usual. Hyposulphites act much like sulphates, as they are quickly reduced in the body to sulphates: sulphites and sulphides are somewhat similar in action, depending partly on the amount of oxidising ferments in the animal. Both sulphates, sulphites, and sulphides cause intense renal irritation; and it is suggested that conceivably they may be, in part at least, the cause of chronic nephritis and cirrhosis of the liver, both frequently ascribed to malted liquors, which are known to contain large amounts of sulphates. These diseases are not common in those whose drinks are free from sulphates, and Dr. Jones suggests that possibly the alcohol in beer has been unjustly blamed for causing cirrhosis of the liver.

### The Diet of the Tsetse.

Observations have been accumulating for some time which show that tsetse flies may thrive in enormous numbers in districts where there is no game—or hardly any—and also that in some districts where game is abundant and conditions seem otherwise suitable to these flies, there may be few or none at all. There are tropical hygienists who believe in the possibility of the tsetse subsisting on a vegetable diet, therefore, and two observations just recorded in the *Sleeping Sickness Bureau Bulletin* by Mr. R. C. F. Maugham support this view. So far Mr. Maugham's experience is unique and unconfirmed; but if confirmation is in time forthcoming from other observers, the problem of the elimination of the tsetse and the diseases which it distributes becomes more complicated and difficult than ever. On one occasion Mr. Maugham saw a common *Glossina morsitans* alight on a stem of marsh grass, insert his proboscis, and suck unmistakably for about two and a half minutes. On the other occasion he was traversing for three days a gameless and almost waterless country; notwithstanding this, *G. morsitans* existed in enormous numbers, and one day he saw one alight on a piece of cut sugar-cane, walk along it to the exposed pulp, and feed there. He is quite positive about the accuracy of these two observations.

### A New Nervous Sign.

A preliminary communication concerning a new diagnostic sign of nervous disease appears in the *Interstate Medical Journal* from the pen of Dr. C. G. Chaddock. He calls it the external malleolar sign, and it consists of extension of one or more or all of the toes, with or without "fanning" of them, when the infra-malleolar skin is irritated on the external side of the foot. The degree of irritation should be varied: in some cases the merest touch is sufficient to excite the sign; in others rather severe scratching may be required. The author is satisfied that such irritation of the external infra-malleolar area causes no reaction normally; that the external malleolar sign is usually present when Babinski's sign is present; that it is often present when Babinski is absent; that it may come before, accompany, and outlast a Babinski's sign; that its presence or absence is a great clinical aid in the interpretation of doubtful or occasional abnormal movements excited from the sole; and, finally, that it denotes disorder of an organic nature in the spino-cortical reflex paths.

### Gastric Motolity and Ingestion of Oil.

At the twenty-eighth German Congress of Internal Medicine Dr. von Tabora read a paper on the effects of ingestion of oil on the peristaltic movements of the stomach. He stated that a dose of 20 to 30 c.c. of oil causes in a few moments a true paralysis of the organ. Gastric motolity reappears in two or three hours, but can be inhibited by further doses of oil. The stoppage of the contractions can be effected as well when fasting as when the stomach is full. It does not, however, prevent evacuation of the stomach, for the pylorus remains open to such a degree that if the patient remains stretched on his right side, food passes in a continuous stream into the duodenum. Thus if small quantities of oil are administered regularly the stomach movements can be stopped for several days on end. This fact is of immense clinical importance, especially in the treatment of gastric ulcer. The oil must be given every two or three hours day and night. Its use also has the advantage of preventing hyperchlorhydria.

### Organic v. Inorganic Iron.

For some years the advantages of iron in organic combination over the inorganic salts of this metal as remedies for anæmia have been freely advertised by the makers of certain proprietary organic iron products. The profession has naturally been somewhat shy of taking for gospel the assertions of interested parties, and many practitioners have continued to prescribe Blaud's pill or modifications of it, or the "scale preparations" of the Pharmacopœia. The independent investigations of Dr. J.

G. M. Munro contributed to the *British Medical Journal* are therefore of value. This author tried the comparative effects of hæmatogen, ferroglidine, Blaud's pill, and citrate of iron and ammonia on mild cases of chlorosis. His experience is that small doses of the organic iron preparations, tested against equivalent doses of the inorganic salts, are absorbed in much greater proportion of the entire amount given. He believes that the large doses of the inorganic salts which are frequently ordered are a mistake, as there is no proof that the economy can make any use of them, whereas smaller doses of the organic salts have just as good an effect without the same disadvantages. He points out that the average iron content of ordinary mixed diet is one-twelfth to one-sixth of a grain, and that this suffices in health to maintain the iron equilibrium.

### Petrol and Benzene as Skin-Disinfectants.

An article by Dr. Zatti, which appears in *Les Annales de Chirurgie*, describes a method of sterilising the skin prior to operation by means of petrol and benzene. The eve of the operation the area of the operation is freed from hair by shaving, but no attempt is made to cleanse the skin by bathing the parts. Next day the patient is placed on the table with a mackintosh sheet under him and a sterilised sheet over him. A large cotton-wool tampon, soaked in petrol so that the liquid does not tend to drip from it, is applied regularly over the operation area for the space of a minute and a half. A similar tampon dipped in commercial benzene is next applied to the same surface for half a minute. The skin is then found to be somewhat oily to the touch, but retains its polish. The operation is carried out forthwith without further preparation. The author has made use of this method of sterilising the skin with the greatest success in a series of over seven hundred operations of all kinds. He claims the following advantages for it: Neither the appearance nor the suppleness of the skin is in any way altered, and no kind of irritation is set up. The skin remains greasy and the blood does not clot over it. Moreover, this form of sterilisation can be applied to any portion of the body.

### Eruptions Due to Antipyrin.

Antipyrin is capable of giving rise to bullous lesions of the buccal cavity, which constitute a variety of stomatitis about which little is at present known, though the condition is of great clinical interest and by no means rare. These eruptions, says L'Huillier in a Thèse de Lyon, are characterised clinically at the onset by pruritus and a red exanthem, followed by bullæ variable in volume, shape, and localisation, which burst, leaving ulcerated surfaces of every conceivable type, aphthous, herpetic, syphiloid, pultaceous, pemphigoid, etc. The evolution of these lesions is rapid, taking from one to fifteen days. It is general for a relapse to take place at each administration of antipyrin, whatever be the dose and the manner of taking it. Some of the rashes are confined exclusively to the buccal cavity, others affect the whole cutaneous surfaces of the body. It is of the utmost importance to discover that the rash is of drug origin, so as to avoid confusing it with one of pathological significance, such as those due to syphilis, true pemphigus, etc., for prognosis and treatment in such cases are entirely different. In the case of an antipyrin bullous eruption the prognosis is good, and all that is necessary to obtain a complete cure is to suppress the drug entirely. It is still uncertain as to how the antipyrin produces these eruptions, though many hypotheses have been advanced to account for the phenomenon. Probably the rash depends on some vaso-motor disturbance effected by the drug.

### Tuberculous Lesions and Radium-therapy.

At a recent meeting of the Paris Academy of Medicine, Dr. Chéron reported the results of a series of investigations carried out by Dr. Dominici and himself with reference to the treatment of deep extra-pulmonary tuberculous lesions by radium-therapy. For the past two years the authors have carried out research into this subject by means of silver, gold, and platinum tubes containing pure radium sulphate, applied in some cases to the surface, in others deep in the tuberculous tissues. The results have been nil in some cases, but more often transitory or permanent improvement has followed, and complete cures have been obtained. It is more particularly in cases of rebellious tuberculous adenopathies, in costal caries of childhood, etc., that the new treatment has proved most efficacious. Cures have commenced after two or three applications of twenty to twenty-four hours each, at monthly intervals. The authors believe in the possibility of curing by radium-therapy certain tuberculous lesions resistant to ordinary treatment, and, without claiming that radium is a specific, they think that it can be made use of with effect as a complement to other physical agents in medicine and surgery.

### Facial Herpes in Scarlet Fever.

Herpes facialis in scarlet fever is by no means uncommon, and its occurrence has been investigated by Dr. J. D. Rolleston (*British Journal of Dermatology*, vol. xxii., No. 10, p. 309). He found it in twenty-seven out of 413 cases of scarlet fever, or 6.5 per cent., which is about the same percentage incidence as that of facial herpes in influenza, and rather greater than that of the eruption in diphtheria—namely, 4.2 per cent. of 1,370 cases. Scarlet fever, therefore, comes fourth in the list of acute infectious disease for which figures of the frequency of herpes facialis are available, following at a long distance pneumonia, malaria, and cerebrospinal meningitis, in each of which it occurs in about 40 per cent. of all cases. Bacteriological examination of the throat did not reveal the predominance of any other organism than the streptococcus, though the possibility of pneumococcal infection was present to Dr. Rolleston's mind at the time.

